# Predation Pressure in Tea (*Camellia sinensis*) Plantations in Southeastern China Measured by the Sentinel Prey Method

**DOI:** 10.3390/insects11040212

**Published:** 2020-03-29

**Authors:** Titus S. Imboma, De-ping Gao, Min-sheng You, Shijun You, Gabor L Lövei

**Affiliations:** 1 State Key Laboratory of Ecological Pest Control for Fujian and Taiwan Crops, Institute of Applied Ecology, Fujian Agricultural and Forestry University, Fuzhou 350002, China; imbomati911@gmail.com (T.S.I.); depinggao@sina.com (D.-p.G.); msyou@fafu.edu.cn (M.-s.Y.); 2 Joint International Research Laboratory of Ecological Pest Control, Ministry of Education, Fuzhou 350002, China; 3 Department of Agroecology, Aarhus University, Flakkebjerg Research Centre, DK-4200 Slagelse, Denmark

**Keywords:** ecosystem services, biological control, landscape effect, edge effects, forest fragments, spillover

## Abstract

Tea (*Camellia sinensis*) is an important food product with thousands of years of human use. Being a non-washable food, no pesticide residues are allowed, which increases the importance of natural means of plant protection. Predation, a component of natural pest control, is an important contributor to this, but its level and sustainability are not known in most of the areas of tea production. We quantified predation intensity using the artificial sentinel prey method in a tea-growing landscape containing remnants of the original forest vegetation in Fujian Province, China. The most common predators were chewing arthropods (49.8% of predation events) and birds (48.1%). Overall, predation rates at the edges of forest fragments (18.9% d^-1^) were lower than either in fragment interiors (25.4%d^−1^) or in the surrounding tea plantations (19.2–24.1%d^-1^). Arthropod predation was higher inside, and at the edge of, forest fragments than within plantations, and generally decreased with increasing distance from a fragment edge, indicating limited spillover of arthropod predators from the native habitat remnants to the cultivated matrix at the local scale. Bird predation, though, showed a different trend: it was lower on the inside of forest fragments than in the tea planation, and bird attack rates increased at increasing distances (up to 40 m) from the forest fragment edge. We also found a reciprocal relationship between attack rates by birds and arthropods, suggesting intra-guild predation. Measures protecting arthropod natural enemies could increase the combined pest suppression effect, contributing to pesticide-free tea production in China.

## 1. Introduction

Ecosystem services (ESs) are based on natural ecological processes, whose outcomes are essential for human existence and well-being [1]. ESs are numerous, from the production of food to the psychological benefits derived from contact with living organisms, and are currently defined under the term “nature’s contribution to people” [2]. There is increased focus on ESs because humankind increasingly depends on them, they cannot be replaced by technology, and in many places of the world, they are under stress, showing signs of damage [3]. 

One of the benefits of natural pest control is the reduction of the densities of organisms considered pests of various crop plants. There are several ecological interactions whose outcome is natural pest control, but predation is undoubtedly an important one among them [4]. There are various means to increase the activity of predators, a major one of which is so-called ‘conservation biocontrol’ (CBC) that seeks to create favourable conditions for naturally occurring biocontrol agents in cultivated landscapes [5]. CBC is a promising approach with notable successes [6], and it is a safe generalisation that there is more potential for CBC in perennial cultures, such as forests, orchards, and tree plantations, than in annual ones [6].

Among perennial cultures, tea (*Camellia sinensis* (L.) Kuntz) occupies a special place. Tea, originally a forest tree, is today cultivated as a shrub or small tree, and a tea plantation is usually kept for several decades, thus constituting one of the longest-living cultivated crop-based habitats. Apart from its importance as a human beverage with a long history of use [7], tea is a very important commodity in China. In 2015, with a production of 2.3 million tons, it accounted for 42.9% of world production; expanding tea cultivation has been important in increasing farmers’ income, and constructing sustainable rural areas [8]. Tea is a ‘non-washable food product’ in which residues, especially pesticide residues, must be carefully checked, and are non-acceptable [9]. This increases the potential and value of minimising agrochemical use during production. Several major pests of tea are arthropods [10]; therefore, biocontrol agents in tea ecosystems could be important. Planting various flowering plants in-between rows of tea in Fujian can increase the abundance of various natural enemies [11], and increase their potential to control pests in tea. 

Although originally a forest tree, today’s tea plantations are still created by converting originally forested habitats to tea gardens. Such and similar conversion of natural areas to agriculture is one of the major drivers of biodiversity loss [12,13]. This conversion process generates landscapes consisting of a ‘matrix’ of cultivated areas, with embedded remnants of natural habitat fragments that vary in number, size, and distance from each other [12]. These habitat fragments serve as refuges for native biodiversity, but also support species providing ESs, such as pest control [14] or pollination [15]. Montane forests had a similar fate: the widespread cultivation of tea, and also of vegetables and floriculture, has led to alarming losses in montane forest cover world-wide [16]. The remaining fragments of the original vegetation can have an increased importance in the CBC of tea pests—however, the impact of those fragments of the original vegetation on this potentially important ES has not been examined nor quantified.

Measuring ESs, however, is not easy. Instead of measuring the function itself, studies often resort to the proxy of densities of natural enemies [17], although this can be misleading [18]. When it comes to predation, the sentinel prey approach is a simple, easy and reliable quantitative method [19]. Given that in Fujian Province, southern China, insects are the most important pests in tea [11], the artificial caterpillar method [20] is suitable for both identifying the main predators active, as well as for a quantitative comparison of the impact of different landscapes on this specific ES [19].

In this work, we quantified predation pressure in a southern Chinese tea-growing landscape, identified the main predators, and examined the relationship between predation pressure and landscape parameters. Our hypotheses were the following:

**Hypothesis** **1 (H1).** Predation pressure in the remaining, original forest fragments is higher than in the surrounding tea plantation. We expected this because the forest fragments have larger standing biomass, and thus more resources for herbivores, which in turn will support more predators, that will also benefit from the lower intensity of disturbance than the regularly cultivated (and thus disturbed) tea garden.

**Hypothesis** **2 (H2).** Predation pressure at the edge is higher than either on the inside of the fragment, or in the surrounding matrix. This can result from several factors and/or their combination. Natural enemies residing at the edges may have access to complementary resources from both adjacent habitats [21], and reach higher densities or activities there [22]. Additionally, the edge can support a specific set of edge-preferring species [23], and the higher predator diversity at these edges may increase predation pressure.

**Hypothesis** **3 (H3).** Predation pressure by arthropod predators decreases with increasing distance from the fragment edge, due to a decrease in the densities or the mobility of natural enemies that reside in the forest fragment and spill over to the surrounding crop [24].

**Hypothesis** **4 (H4).** Birds will not show a similar gradient in predation pressure by increasing distances from the nearest forest patch, because they have higher mobility.

Our results provided mixed support for the above hypotheses: arthropod predation was indeed higher inside forest fragments (H1 partially supported), even higher than at the edge (H2 not supported), and attack rates on sentinel prey indeed decreased with increasing distances from the forest edge (H3 supported). Birds did not show such a decrease, and their predation pressure was higher in tea plantation than inside forest fragments (H4 supported). 

## 2. Materials and Methods 

### 2.1. Study Sites

The study sites were in Fujian Province, in Southern China. Fujian is one of the main tea-producing regions of the country, with strong historical traditions and a mountainous landscape with a humid subtropical climate. 

**Figure 1:**
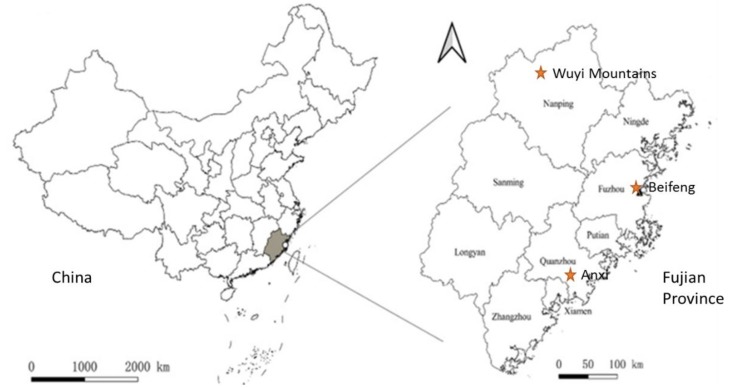
The geographical locations of the three sampling sites (Wuyi Mountains, Beifeng and Anxi) in Fujian Province, Southeastern China.

The study was performed at three sites within Fujian Province (Figure 1): in the Wuyi Mountains in the north-west (N27˚42’ E117˚56’, 231–375 m asl), at Beifeng (N26˚ 10’, E 119˚ 23’, 575 m asl) in the central highlands, and at Anxi in the Southeastern part of the province (N24˚ 57’, E 117˚ 49’, 814 m asl).

At Wuyi, the tea farms of the Red Star Company were used for the experiments. Wuyi Red Star is one of the major producers of organic oolong tea, with 1300 ha of plantations of >10 different cultivars. The gardens are divided into two blocks, located just outside Wuyi city. One block was established 5 years ago, with regularly planted indigenous native trees, shrubs and herbs between the rows of tea. These were planted to provide shade to the tea crop, as well as cover for wildlife including arthropods, reptiles, birds and mammals. The most common intercropped tree species included the evergreen shrub *Lirianthe championii*, fragrant olive *Osmanthus fragrans*, *Eucaphis japonica* (the most common tree), the Korean sweetheart tree *Eurya japonica*, the camphor tree *Cinnamomum camphora*, as well as neem *Melia azadarichna*, black chokeberry *Photinia prunifolia*, the chinquapin tree *Castanopsis chinensis,* and *Photinia davidsonia*. This site had, at its borders, single-lane rows of exotic pines, whose undergrowth was a dense stand of native ferns and various shrubs. The second block was about 20 years old, with much older lines of planted trees as well as remnant patches of the original forest vegetation. This site was not intercropped with trees. Both farms were organically managed. Tea was machine-harvested and pruned twice a year. In places that were too steep to clear for planting tea, smaller or bigger (up to 6 ha) fragments of the original forest were left. The forest fragments had trees of up to 30-m tall, with dense bamboo growing near creeks or watercourses, and a dense bush layer of up to 8m. The trees included the Himalayan yew, *Taxus wallichiana*, neem, *Abies holophylla*, *P. davidsonia, E. japonica, Ormosia henryi,* and *Podocarpus macrophyllus.* The dense shrub layer contained several species, among them *Loropetalum chinense, P. prunifolia, Rhododendron simsi, Eu. japonica, Koelreuteria paniculata*. 

Anxi County, with an approximate area of about 20,000 ha of tea plantations, is the highest producer of tea in Fujian Province. The government initiative for the spatial agglomeration of tea gardens resulted in a landscape of continuous tea gardens, which, although belonging to different farmers, spanned from the foot of the mountain to the top without physical boundaries. These were composed of undocumented mixed cultivars, and managed using different gardening techniques including intercropping tea with indigenous trees, and organic farming using livestock manure while other owners used synthetic chemical fertilizers. The landscape was more varied here, including unmanaged edges of natural grasses and woody vegetation, as well as patches of exotic pine forests and fragments of natural forests. The pine forest was of *Pinus armandi*, with a dense understory of shrubs and ferns. The natural forests had *Podocarpus sinensis, Prunus canisifera,* and individuals of *Salix babylonica.* A prominent climber was *Clemantis finetiana*. We selected organically managed tea plantations belonging to Juyuan Tea Professional Cooperative. 

The Beifeng site belonged to the Ecological Chunlun Tea Gardens, part of the 1200 ha of organic jasmine tea production area in Fujian Province. This site had discontinuous small strips and island-like patches of natural and pine forests, as well as natural grasses including bamboo. The tea plantations were of mixed cultivars, organically managed, and tea was hand-picked twice yearly. Free ranging cows and goats were grazed on the natural grasses at the edges, and also in the plantation to suppress weeds. The forest fragments were similar in physiognomy to the Anxi site, with *Cerasus serulata, Elaeocarpus sylvestris, Machilus nanmu* and *Cinnamomum burmanni*, while the shrubs included *Acer palmatum, Photinia. frasseri, Osmanthus mastsuranus* and *Pyrenara spectabilis.*

### 2.2. Artificial Caterpillars 

In order to quantify predation pressure, we used the sentinel prey method using artificial caterpillars, following the best practice as suggested by Low et al. [25] and Lövei and Ferrante [19]. Artificial caterpillars were made of non-drying green plasticine (SmeedyPlus, Viborg, Denmark), were 15-mm long, and 3 mm in diameter. The colour green was chosen because it is perceived to be palatable by most predators [20]. At each location, two sites were selected, at least 200 m from each other. At one site, five positions were chosen: inside a remnant forest patch, at the edge of the patch, and in the tea plantation at 5m, 20m, and 40 m from the forest patch’s edge. At each position, 10 caterpillars were glued with a drop of superglue (Loctite®) onto a leaf of a tea plant or a suitable bush in the undergrowth. Individual caterpillars were 5m from each other, and the line of caterpillars ran parallel to the forest edge. Thus, a total of 50 caterpillars were used in each site during one census occasion. The prey were left on site for 24 h before they were observed for signs of attack using a hand-held magnifying lens (10 x magnification), photographed, and, if necessary, carefully removed for further identification in the laboratory. Experiments were performed at Anxi between September 5, 2018 and July 20, 2019 (10 sessions); at Wuyi between August 9, 2018 and July 27, 2019; and at Beifeng between September 13, 2018 and July 9, 2019 (8 sessions each). Ten caterpillars (0.77%) were not possible to recover.

### 2.3. Data Evaluation

The analyses were carried out and graphs were created using the statistical software R [26] through Rstudio [27]. We defined three generalised linear mixed models (GLMM) with binomial distribution and log-link function to analyse separately overall, arthropod, and bird predation rates. The fixed covariates in the models were location (Anxi, BeiFeng, and Wuyi), habitat (forest, edge, 5 m, 20 m, and 40 m from the edge), sampling period (summer 2018, spring 2019, and summer 2019), and the interaction between habitat and sampling period, while site (2 sites at each location) was considered as a random factor. For each predatory group, we determined the importance of the fixed covariates comparing Akaike Information Criterion values [28]. As all covariates consisted of categorical factors with more than two levels, important factors retained in the best models were further tested using the Tukey’s post-hoc test, using the *lsmeans* package [29].

## 3. Results

### 3.1. Overall Predation Pressure

Out of a total of 1300 dummy caterpillars glued onto tea canopies (400 each in Wuyi and Beifeng, 500 at Anxi), 283 caterpillars were found with predation marks. This represents an overall attack rate of 21.94% within the 24 h of exposure. The major predators were arthropods (n = 141, 10.93% d^−1^), and birds (n = 136, 10.54% d^−1^), with a few reptiles (n = 3, 0.23% d^−1^), and a single attack by a small mammal; two were made by unknown predators. 

Wuyi had the highest attack rates (22.47%d^−1^), with arthropods causing most of the predation, followed by birds, and one mammal (Table 1). Beifeng had a total of 87 attacked caterpillars (22.3% d^−1^). Only arthropods and birds were recorded predators (Table 1). Anxi recorded an attack rate of 21.44% d^−1^; this location had 58 caterpillars attacked by birds, 44 by arthropods, and three by unidentified predators. 

### 3.2. Seasonal Variation in Predation Pressure

The three seasons showed inconsistent changes in predation rates, varying between 19.0% d^−1^ and 25.6%d^−1^ (Table 1). The overall predation pressure was higher (but not significantly so, *p* = 0.136–0.681) in both summers than in spring 2019, but the relative strength of predation pressure by arthropods vs. birds was different, especially between the two summers (Table 1).

### 3.3. Predation Pressure in Forest Fragment vs. Tea Plantation

**Figure 2:**
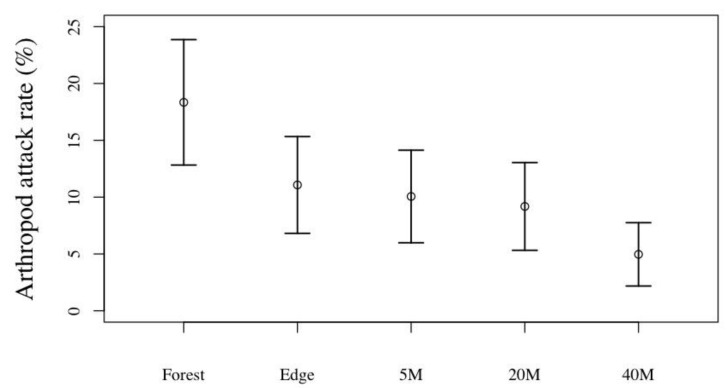
Mean attack rates on artificial caterpillars by arthropods in forest fragments and on tea plantations, at various distances from the forest, at three tea-growing regions (Wuyi, Beifeng, Anxi) in Fujian, during spring and summer.

The overall attack rates did not show a clear trend from the forest fragment interior towards the centre of the tea plantation (Table 1). This was, however, due to the different spatial trends by the two most important predator groups. Arthropods were the most frequent attackers of the caterpillars in forest fragments (Table 1), and this gradually decreased from the forest edge towards the plantation centre. The most parsimonius model indicated a significantly higher predation pressure in the forest than on the tea plantation; even at the edge, attack rates were lower, but not significantly so (Figure 2). 

**Figure 3:**
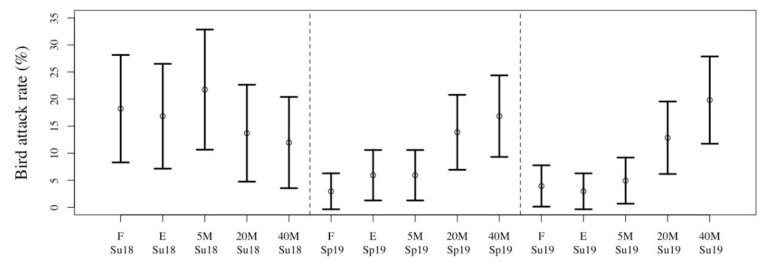
Attack rates on artificial caterpillars by birds, in forest fragments and on tea plantations, at various distances from the forest, in three tea-growing regions (Wuyi, Beifeng, Anxi) in Fujian, during spring and summer. Codes: Su18: summer, 2018; Sp19: spring 2019; Su10: summer 2019. F: forest fragment interior; E: forest fragment edge; 5M, 20M, 40M: distance (in m) from the fragment edge.

Predation by birds showed an opposite trend: it was lowest in the forest, and increased gradually from the forest edge into the tea plantation (Table 1). The general linear models indicated that study location was not an important factor influencing attack rates, but position (in relation to the forest fragment) and seasons were influential. There was no clear spatial trend during summer 2018, while both spring and summer 2019 showed that birds were more active away from the forest edge than inside (Figure 3). Indeed, fewer birds were recorded in the forest, and the number increased from the forest edge towards the center of the tea plantation (T. Imboma, personal observation).

**Figure 4:**
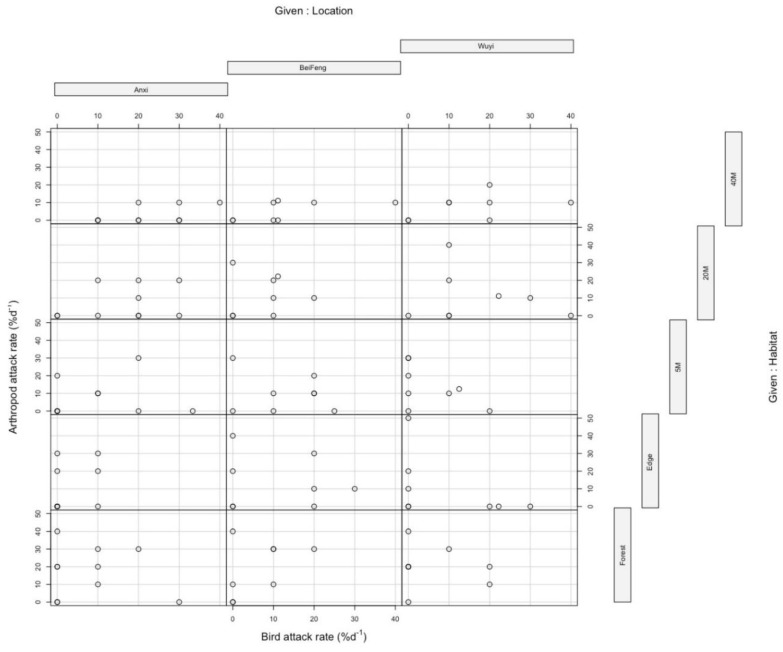
The relationship between predation by birds and arthropods at various locations in tea plantations, in three tea-growing regions of Fujian Province, southeastern China.

The relationship between arthropod vs. bird predation often showed a negative relationship (Figure 4): arthropod (mostly insect) attacks were generally higher in the forest than away from it, while bird attack rates showed an opposite trend. In several locations there was also a reciprocal relationship between the activity of the two major predator groups: where bird attack rates were high, insect attack rates were low and vice versa (Figure 4).

## 4. Discussion

Our results constitute the first quantitative study on predation pressure in tea, and add to the still-sparse knowledge of predation pressure in cultivated habitats in China [19], as well as world-wide [30]. We documented that several groups of natural enemies, including birds associated with forest vegetation, regularly visited tea plantations and attacked arthropod prey encountered there. The latest summary of published data on attack rates on artificial caterpillars in cultivated areas world-wide [30] shows maximum arthropod attack rates of about 10%d^-1,^ with no latitudinal gradient. The rates documented here (5.0% d^–1^–10.1% d^–1^) fall near the highest values recorded so far. This is even more remarkable when we consider that we measured predation pressure on vegetation, while many of the existing data were collected at ground level. Attack rates on sentinel prey exposed on vegetation are usually lower than at ground level [30]. 

Birds are important predators in tropical agroforestry systems [31]. Vertebrate attacks on artificial caterpillars are mostly by birds, and predation pressure is typically higher than by invertebrates [19]. Attack rates on artificial caterpillars by birds in tea plantations in Fujian (up to 17.0% d^−1^) were also in the higher range; most existing data are below 10% d^−1^ [30]. 

We also demonstrated that birds ranged farther into tea plantations than arthropod predators did, with the latter showing limited spillover from forest fragments. Spillover effects can be strong, as was found for pest control and pollination in Britain [32], but not always: weak or non-existent spillover was documented from cultivated areas in Central Europe [33], and South America [34]. Different organisms perceive the landscape differently, depending on their habitat specialisation, tolerance limits, dispersal power, or feeding habits [35]. There is also a potential seasonal variation [33] which we were unable to track. Spillover is also very likely influenced by the nature of edges [36]. Apparently, in our study system, the edges had a deterrent effect on arthropods active in forest fragments. 

The reciprocal relationship between arthropods and birds has not been previously reported elsewhere. This type of intraguild predation can be explained by birds preying on several groups of arthropod predators [37]. Intraguild predation is not an infrequent phenomenon, and occurs between vertebrate and arthropod predators, as well as between various groups of invertebrates, for example between carabids and spiders [38]. Ground beetles are an important group of generalists, and carabid activity density can be positively correlated with the attack rates on artificial caterpillars [39]. Small mammals can also be predators of carabids, decreasing their densities [40], and possibly reducing the overall predation pressure by natural enemies. However, in our study site, carabids were not a dominant group (G. Pozsgai, FAFU, Fuzhou, personal communication), and they are mostly night-active [38]; thus, one would not expect such a reciprocal relationship if carabids were responsible for most of the attacks on our caterpillars. It is plausible to assume that most of the arthropod attacks were made by day-active predators, such as ants and spiders, that are common in these habitats [11], and these would be directly threatened by invertivorous birds looking for food. It was also clear that at least some bird species were more active on the tea plantation than in the forest fragment (T. Imboma, personal observation).

## 5. Conclusions

In conclusion, the recorded high attack rates on artificial caterpillars were promising, indicating a relatively high predation pressure, both by birds and arthropods. The reciprocal relationship between these two groups hinted at intra-guild predation, where birds would be the intraguild predator, and arthropods the intraguild prey. Measures to protect arthropod natural enemies, e.g. by providing shelter for them at ground level, and by planting flowering crops and permanent ground cover, could increase their density [10], boost their impact on their prey, and protect them from intraguild predators. This could allow an increased combined pest suppression effect, and would be a useful step towards pesticide-free tea production in China.

## Figures and Tables

**Table 1:** Summary statistics of the attack rates on artificial caterpillars placed on vegetation in three tea-growing regions in Fujian Province, China. Data are means ± S.D. (n).

Location/Season/ Habitat	Attack Rates (% d^−1^) by		
	All Predators	Arthropods	Birds
Wuyi	23.0 ± 14.9 (40)	12.3 ± 13.1 (40)	10.4 ± 11.8 (40)
Beifeng	22.0 ± 16.0 (40)	11.8 ±12.4 (40)	10.2 ± 10.0 (40)
Anxi	21.5 ± 17.1 (50)	8.8 ±11.5 (50)	11.7 ± 11.6 (50)
Summer 2018	25.6 ± 12.2 (30)	8.7 ±10.0 (30)	16.6 ± 9.6 (30)
Spring 2019	19.0 ±17.3 (50)	9.8 ±12.0 (50)	9.2 ± 11.8 (50)
Summer 2019	23.0 ± 16.3 (50)	13.2 ±13.6 (50)	9.0 ± 10.4 (50)
Forest	25.4 ± 16.3 (26)	18.5 ±13.5 (26)	6.9 ± 8.8 (26)
Forest edge	18.9 ± 13.6 (26)	11.2 ±14.8 (26)	7.4 ± 10.5 (26)
Tea plantation, 5m	19.4 ± 13.6 (26)	10.1 ±11.0 (26)	9.3 ± 10.1 (26)
Tea plantation, 20m	24.1 ± 16.2 (26)	9.4 ± 11.4 (26)	13.6 ± 11.0 (26)
Tea plantation, 40m	22.8 ± 16.3 (26)	5.0 ± 5.9 (26)	17.0 ± 12.2 (26)
